# Novel adherent CD11b^+^ Gr-1^+^ tumor-infiltrating cells initiate an immunosuppressive tumor microenvironment

**DOI:** 10.18632/oncotarget.24359

**Published:** 2018-01-29

**Authors:** Takuya Tsubaki, Tetsuya Kadonosono, Shimon Sakurai, Tadashi Shiozawa, Toshiki Goto, Shiori Sakai, Takahiro Kuchimaru, Takeharu Sakamoto, Hitomi Watanabe, Gen Kondoh, Shinae Kizaka-Kondoh

**Affiliations:** ^1^ School of Life Science and Technology, Tokyo Institute of Technology, Yokohama 226-8501, Japan; ^2^ Division of Molecular Pathology, Institute of Medical Science, The University of Tokyo, Minato-ku, Tokyo 108-8639, Japan; ^3^ Laboratory of Integrative Biological Science, Institute for Frontier Life and Medical Sciences, Kyoto University, Kyoto 606-8507, Japan

**Keywords:** myeloid-derived suppressor cells, cancer microenvironment, tumor immunology, angiogenesis, tumor-associated macrophage

## Abstract

The immunosuppressive tumor microenvironment is a hallmark of cancer. Myeloid-derived suppressor cells (MDSCs) are CD11b^+^ Gr-1^+^ tumor-infiltrating immature myeloid cells that strongly mediate tumor immunosuppression. The CD11b^+^ Gr-1^+^ cells are a heterogeneous cell population, and the impacts of each subpopulation on tumor progression are not yet completely understood. In the present study, we identified a novel subpopulation of CD11b^+^ Gr-1^+^ cells from murine lung carcinoma tumors according to their strongly adherent abilities. Although strong adherent activity is a unique property of macrophages, their marker expression patterns are similar to those of MDSCs; thus, we named this novel subpopulation MDSC-like adherent cells (MLACs). Unlike known MDSCs, MLACs lack the ability to suppress cytotoxic T lymphocytes and differentiate into tumor-associated macrophages (TAMs), but could still directly facilitate tumor growth and angiogenesis through secreting CCL2, CXCL1/2/5, PAI-1, MMPs, and VEGFA. Furthermore, MLACs recruited MDSCs via the secretion of CCL2/5 and CXCL1/2/5, thereby enhancing the immunosuppressive tumor microenvironment and promoting TAMs-mediated tumor progression. Our findings suggest that MLACs may function as an initiator of the immunosuppressive tumor microenvironment and highlight a new therapeutic target to prevent the onset or delay malignant progression.

## INTRODUCTION

A solid tumor comprises various types of stromal cells such as endothelial cells, fibroblasts, and immune cells [1]. Among them, myeloid-derived suppressor cells (MDSCs) are one of the key drivers of the immunosuppressive tumor microenvironment that is a hallmark of cancer [2, 3], and several therapeutic strategies targeting MDSCs are currently under clinical trials [4].

In mice, MDSCs were historically defined as cells expressing both the typical myeloid lineage marker CD11b and the granulocytic marker Gr-1 [5]. These cells have been found in the peripheral blood, spleen, and lymph nodes of various types of tumor-bearing hosts [6–10]. MDSCs represent heterogeneous populations of myeloid cells and are generally divided into two major subsets in mice: CD11b^+^ Gr-1^lo^ Ly6G^−^ Ly6C^hi^ monocytic MDSCs (Mo-MDSCs) and CD11b^+^ Gr-1^hi^ Ly6G^+^ Ly6C^lo^ polymorphonuclear MDSCs (PMN-MDSCs) [11]. Three main mechanisms by which MDSCs promote tumor progression have been proposed. First, MDSCs induce angiogenesis via secreting VEGFA and MMP-9, which in turn promote tumor growth [12]. Second, MDSCs suppress the activities of cytotoxic T lymphocytes (CTLs) [11]. PMN-MDSCs inhibit CTLs through the increased activity of NADPH oxidase [13], leading to the production of reactive oxygen species, which suppresses the expression of T-cell receptor (TCR) and IFN-γ [14]. Alternatively, Mo-MDSC-induced CTL suppression is strongly dependent on inducible nitric oxide synthase (iNOS) and arginase 1 (Arg1) [11]. The nitric oxide produced by iNOS then inhibits T cells by nitrating TCRs [15], and Arg1 metabolizes L-arginine to L-ornithine, leading to the suppression of T cell proliferation and TCR expression because of the shortage of L-arginine in the tumor microenvironment [16]. Finally, both Mo-MDSCs and PMN-MDSCs differentiate into tumor-associated macrophages (TAMs) [11], which facilitate tumor cell growth and survival via secreting IL-6, EGF, and TNF [17, 18] and promote angiogenesis by VEGFA, Semaphorin 4D, and IL-8 [18–20]. TAMs also suppress CD8^+^ T cell functions by secreting Arg1, TGF-β, and IL-10, and by inducing the expression of the ligands of the inhibitory receptors programmed cell death protein 1 (PD-1) and cytotoxic T-lymphocyte antigen 4 (CTLA-4) on the cell surface [18, 21–23].

A recent study showed that a portion of the CD11b^+^ Gr-1^+^ cells collected from a tumor tissue did not undergo differentiation into TAMs and dendritic cells (DCs) based on an *in vitro* differentiation assay [11]. Furthermore, CD11b^+^ Gr-1^+^ cells isolated from the premalignant lung tissue of a mouse model of spontaneous lung cancer were unable to suppress CTLs [24]. These findings suggest that CD11b^+^ Gr-1^+^ cells may represent an as-yet-undefined subpopulation of MDSCs. To further support this possibility, in the present study, we isolated a novel CD11b^+^ Gr-1^+^ subpopulation and examined the role of these cells in tumor biology and the generation of the immunosuppressive tumor microenvironment using a mouse model and a variety of cancer cell lines. The present characterization of these novel cells should contribute new insight into the mechanisms of host immunosuppression and tumor malignancy and highlight new therapeutic strategies for improving cancer treatment.

## RESULTS

### MDSC-like adherent cells are novel tumor-infiltrating myeloid cells

**Figure 1:**
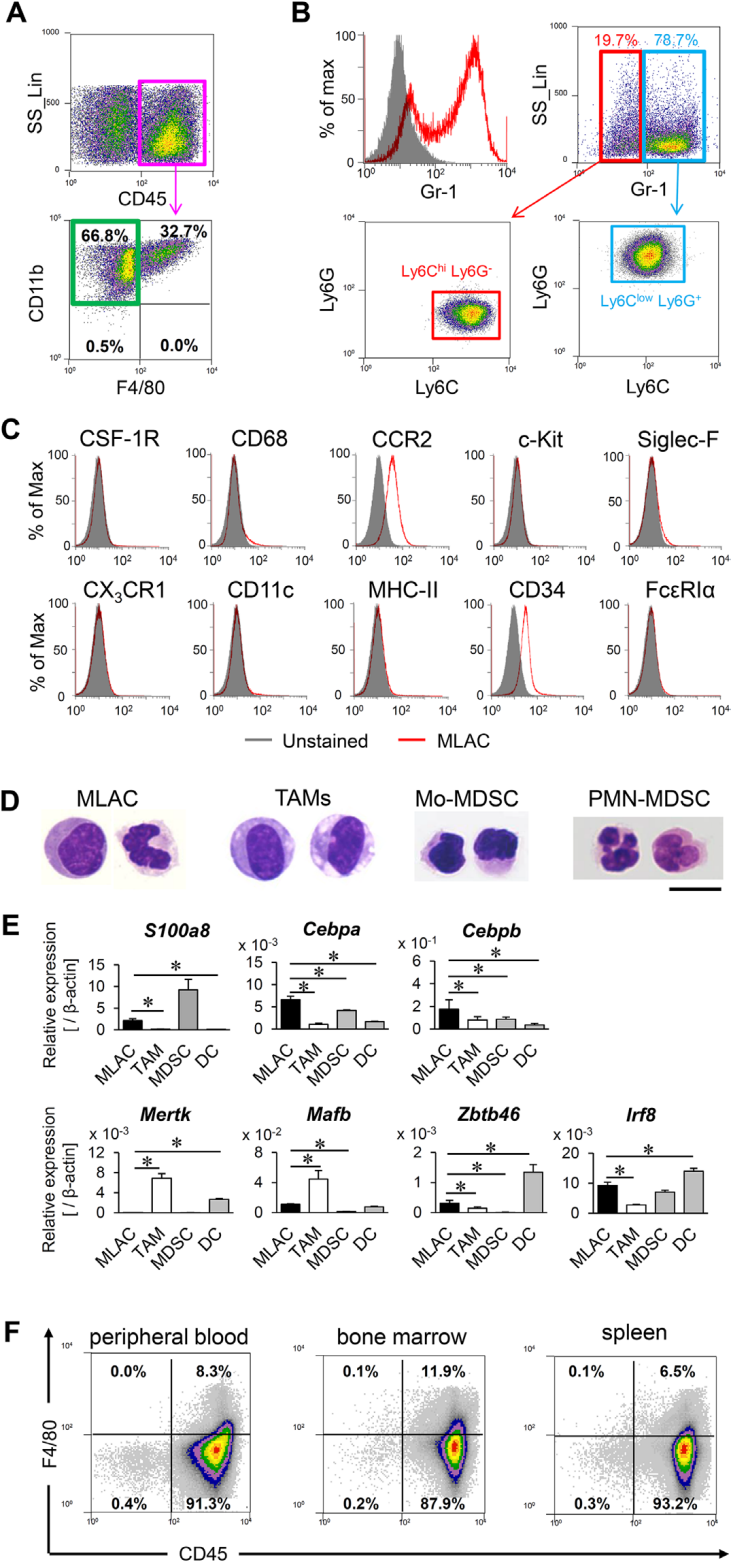
MLACs are novel tumor-infiltrating myeloid cells **(A)** Flow cytometric analysis of adherent cells collected from subcutaneous tumors. The CD45^+^ adherent cell fraction (magenta square) were analyzed for expression of CD11b and F4/80. **(B)** The CD11b^+^ F4/80^−^ adherent cells were analyzed for Gr-1 expression (red histogram). Gray-filled histogram indicates negative control (unstained cells). The Gr-1^hi^ (blue square) and Gr-1^low^ (red square) fractions were further analyzed for expression of Ly6C and Ly6G. **(C)** Marker expression on MLACs. Expression of indicated markers on MLACs were shown by red histograms. Gray-filled histograms indicate negative controls (unlabelled cells). **(D)** Representative May-Grunwald Giemsa stained images of MLACs, TAMs, PMN-MDSCs, and Mo-MDSCs. Scale bar: 10 μm. **(E)** Transcript levels of myeloid cells marker genes in MLACs, TAM, MDSC, and DC. DC represents BMDC. Indicated gene expressions were examined by qRT-PCR. Error bars indicate SEM; ^*^, *P*<0.05 vs. MLACs. *n =* 3. **(F)** The presence of MLACs in normal tissues of tumor-bearing mice. Adherent cells were collected from peripheral blood, bone marrow, and a spleen when a subcutaneous tumor reached 15-20 mm in diameter. All the experiments were performed at least three times and representative results are shown.

In order to study MDSCs in tumors, murine lung carcinoma LLC cells were subcutaneously transplanted into mice, and CD11b^+^ Gr-1^+^ cells were isolated from tumor-infiltrating cells expressing the common leukocyte antigen CD45. When these cells were cultured on a dish, some cells were strongly attached to plastic surfaces. Because the adherent phenotype is a unique property of macrophages [25] and TAMs represent a prominent component of the infiltrating leukocytes in most malignant tumors [26], we thought at first that these were contaminating macrophages. Therefore, we examined the expression of F4/80, a widely used marker for monocytes and macrophages [27]. However, a majority of the cells were unexpectedly negative for F4/80. To confirm the presence of a CD11b^+^ Gr-1^+^ F4/80^−^ adherent cell population in tumors, the cells isolated from subcutaneous LLC tumors were cultured on dishes to select for strongly adhering cells. Among the cells expressing CD45, those showing the strongest adherence were further assessed for expression of CD11b and F4/80; more than half of the CD11b^+^ cells were negative for F4/80 (Figure 1A, green squares). These CD11b^+^ F4/80^−^ cells consisted of both Gr-1^lo^ Ly6C^hi^ Ly6G^−^ and Gr-1^hi^ Ly6C^lo^ Ly6G^+^ cell populations (Figure 1B), corresponding to the characteristics of Mo-MDSCs and PMN-MDSCs, respectively [28]. The CD11b^+^ Gr-1^+^ F4/80^−^ cells did not express monocyte markers (CD68, CX3CR1) or the markers of DCs (CD11c), mast cells (c-Kit) [29], eosinophils (Siglec-F) [30], or basophils (FcεRIα) [31] (Figure 1C, Supplementary Table 1), and they only weakly expressed CCR2 and the hematopoietic progenitor cell marker (CD34) (Figure 1C).

Cell morphological analysis revealed that the CD11b^+^ Gr-1^+^ F4/80^−^ cells did not contain granules such as those observed in eosinophils and basophils [32] but showed similarity to MDSCs with respect to the violet-stained cytoplasm and nuclear shape (Figure 1D). In addition, MDSC subsets generally lack F4/80 expression (Supplementary Table 1). Quantitative RT-PCR (qRT-PCR) analysis of mRNA levels among myeloid-derived cells revealed that the genes representative of immature myeloid cells (*S100a8*, *Cebpa, Cebpb*) [33, 34] were expressed at similar levels in the CD11b^+^ Gr-1^+^ F4/80^−^ cells and MDSCs, and the CD11b^+^ Gr-1^+^ F4/80^−^ cells showed only modest expression of the marker genes of TAMs (*Mertk, Mafb*) [35] and DCs (*Zbtb46, Irf8*) [33, 36] (Figure 1E). The results of morphology and marker gene expression analysis collectively indicated that the CD11b^+^ Gr-1^+^ F4/80^−^ adherent cells are novel immature myeloid cells that are more similar to MDSCs than to macrophages. Therefore, we named this new cell population MDSC-like adherent cells (MLACs). These MLACs were detected in the peripheral blood, bone marrow, and spleen of tumor-bearing mice (Figure 1F). By contrast, no similar adherent cells were obtained from these same organs of naive mice using the same method of detection performed for the tumor-bearing mice. This difference suggested that MLACs, similar to MDSCs, would be specifically generated in tumor-bearing animals.

### MLACs have distinct tumor-promoting effects from MDSCs

**Figure 2:**
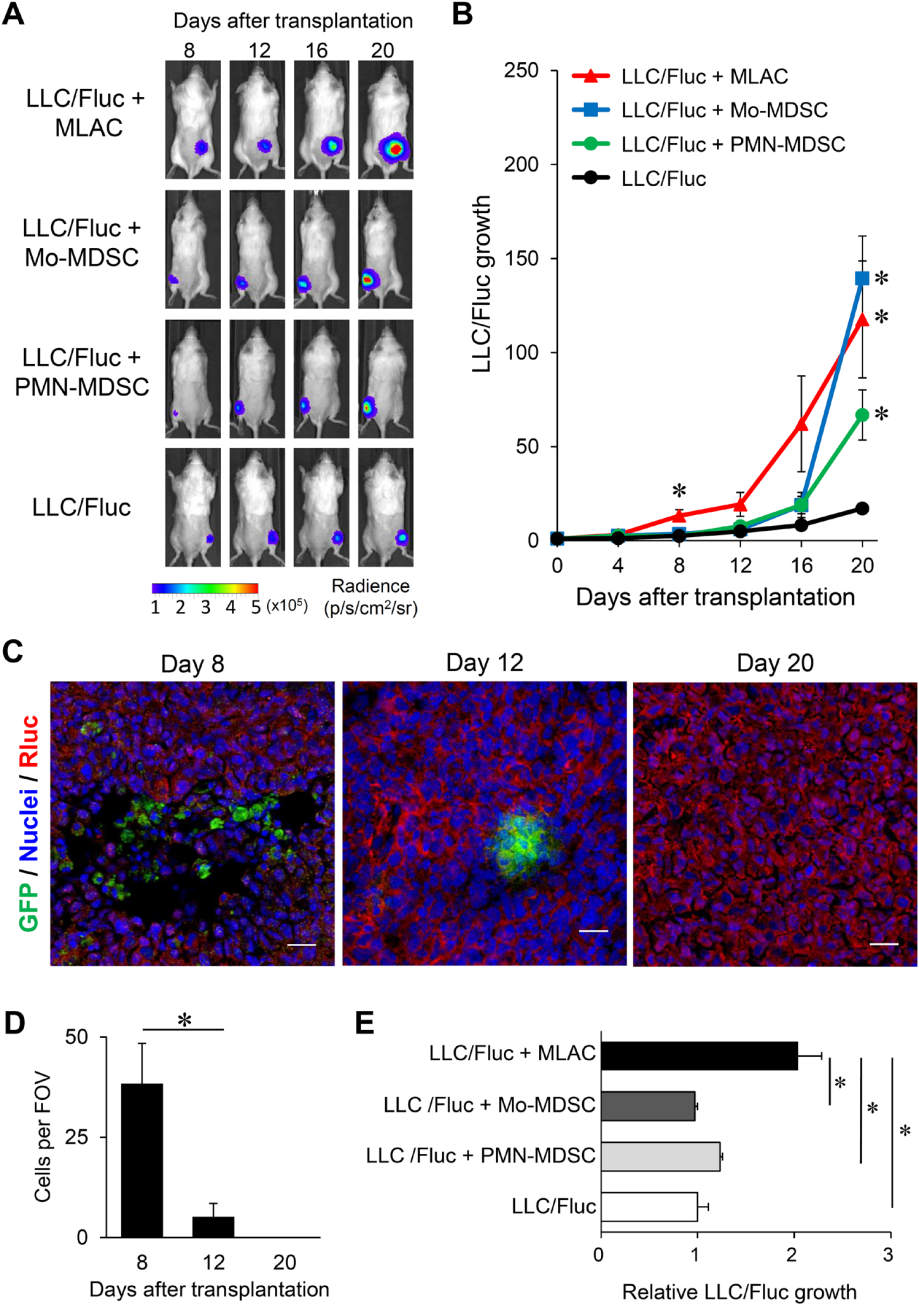
MLACs have distinct tumor-promoting effects from other myeloid-derived cells **(A, B)** Tumor growth-promoting activity of MLACs, Mo-MDSC, and PMN-MDSC. (A) Representative bioluminescence images of the subcutaneous tumors 8, 12, 16, and 20 days after cell transplantation. (B) Quantitative analysis of bioluminescence intensities of tumors in A. LLC/Fluc growth represents the relative BL intensity of each tumor compared to the one at day 0. Error bars indicate SEM; ^*^, *P*<0.05 vs. LLC/Fluc. *n =* 6; LLC + MLAC, *n =* 4; LLC + Mo-MDSC, *n =* 4; LLC + PMN-MDSC, *n =* 8; LLC. **(C, D)** Lifetime analysis of MLACs. MLACs were isolated from LLC subcutaneous tumors of GFP-Tg mice, and subcutaneously co-injected with LLC/mKO2-Rluc8.6 to B6 albino mice. Subcutaneous tumors were resected on days 8, 12, and 20 after co-injection. GFP (green) and Rluc (red) were detected by immunofluorescence staining of tumor cryosections. Nuclei are indicated by blue. Representative immunofluorescence staining images of the LLC tumors (C) and the average number of GFP-positive donor MLACs of 5 fields of view (FOV) (D) are shown. Scale bar: 20 μm. **(E)** Direct growth-promoting effects of MLACs, Mo-MDSC, and PMN-MDSC on LLC. LLC/Fluc cells were co-cultured with MLACs, Mo-MDSC, or PMN-MDSC in a chamber with a membrane insert. After 48 hr of co-culture, luciferase activity of LLC/Fluc cells was measured. Relative LLC/Fluc growth represents the relative BL intensities of co-cultured LLC/Fluc compared to the one of mono-cultured LLC/Fluc. Error bars indicate SEM; ^*^, *P*<0.05 vs. LLC/Fluc mono-culture. *n =* 4. All the experiments were performed at least three times and representative results are shown.

The comparative effect of MLACs and MDSCs on tumor growth was investigated using a tumor mouse model in which LLC cells expressing firefly luciferase (LLC/Fluc) were subcutaneously transplanted along with MLACs or MDSCs into syngeneic mice, and their growth was monitored by *in vivo* bioluminescence imaging (Figure 2A). Although both MLACs and MDSCs significantly promoted LLC tumor growth, the tumor-promoting function of MLACs was apparently distinct from that of MDSCs. The time course of tumor promotion by MLACs exhibited two phases: an early phase at around day 8 and a late phase at around day 20, whereas MDSCs only significantly promoted LLC tumor growth in the late phase (Figure 2B).

To further investigate the tumor-promoting activity of MLACs at the early phase, we first analyzed the life span of these cells after subcutaneous transplantation. The MLACs isolated from an EGFP(K268Q)-GPI transgenic (GFP-Tg) mouse [37] were co-transplanted with LLC cells expressing *Renilla* luciferase fused with the monomer KusabiraOrange2 (LLC/mKO2-Rluc8.6), and the presence of GFP^+^ MLACs in tumor sections prepared on day 8, 12, and 20 after transplantation was examined. As shown in Figure 2C and 2D, a large amount of GFP^+^ MLACs was detected in the tumors on day 8 but not on days 12 and 20, indicating that the transplanted MLACs would function in tumors for at least 8 days after transplantation. These results strongly suggest that the tumor growth enhancement in the early phase would reflect any direct effects of the transplanted MLACs.

To verify the direct effects of MLACs on the growth of LLC cells, the MLACs were co-cultured with LLC/Fluc cells with membrane inserts for 48 hr, and LLC cell growth was evaluated by measuring the bioluminescence intensity. The results showed that the MLACs significantly facilitated the growth of co-cultured LLC cells (Figure 2E), confirming their direct effects on LLC cells. These results clearly demonstrated that MLACs have tumor-promoting effects distinct from those of MDSCs.

For more detailed analysis of the tumor-promoting activity of MDSCs *in vivo*, we also examined the life spans of these cells after subcutaneous transplantation. The Mo-MLACs and PMN-MDSCs isolated from GFP-Tg mice [37] were separately co-transplanted with LLC/mKO2-Rluc8.6, and tumor sections prepared 8, 12, and 16 days after transplantation were analyzed for GFP^+^ MDSCs. As shown in Supplementary Figure 2, a large number of GFP^+^ MDSCs were detected in the tumors on days 8 and 12 but not on day 16, indicating that the transplanted MDSCs functioned in tumors for at most 15 days after transplantation. These results strongly suggest that the tumor growth enhancement in the late phase reflects immunosuppressive effects induced by the transplanted MDSCs.

### MLACs promote tumor growth via CXCL1/2/5/CXCR2 signaling

**Figure 3:**
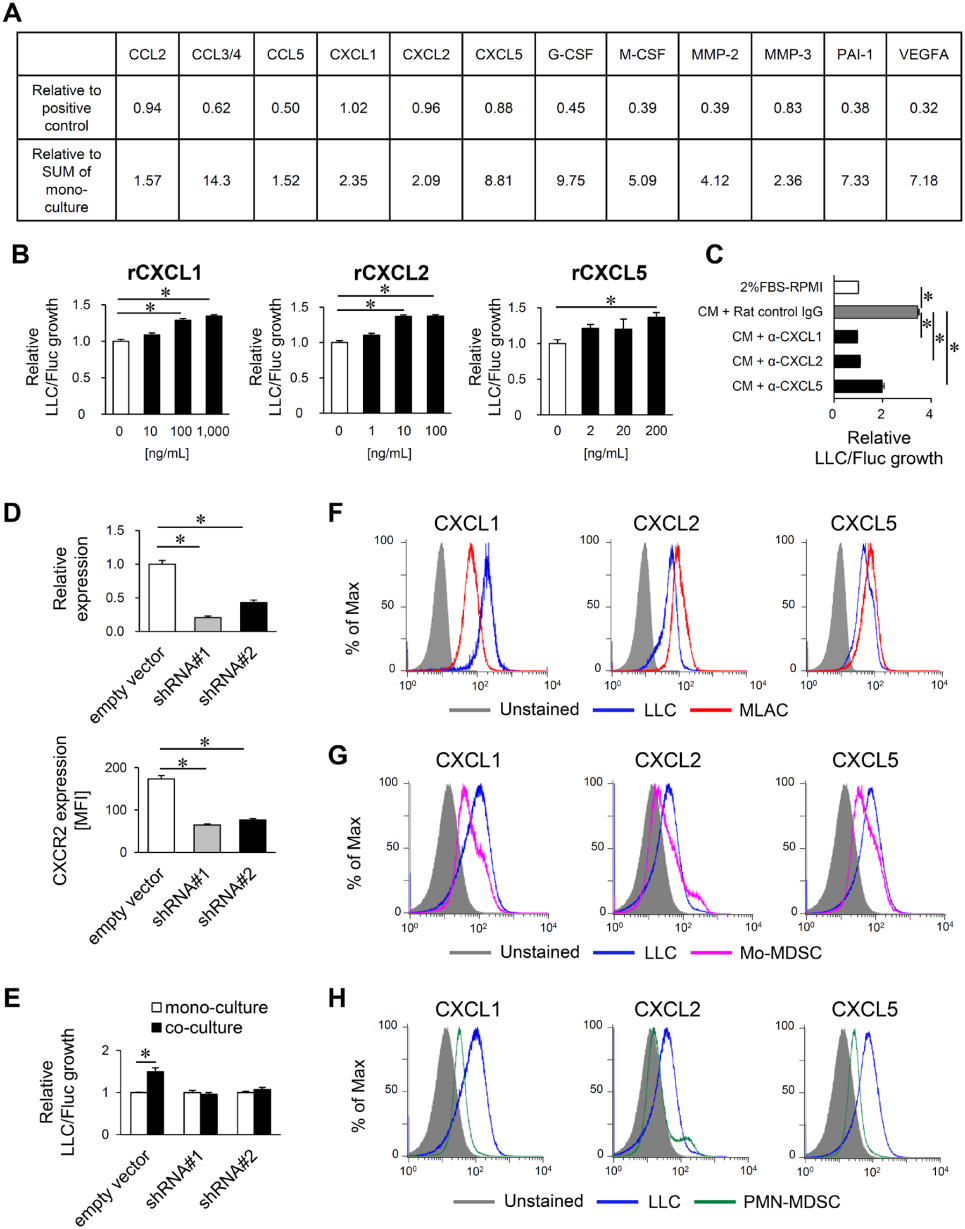
MLACs promote tumor growth via CXCL1/2/5/CXCR2 signaling **(A)** List of soluble factors markedly increased in the co-culture medium of MLACs and LLC/Fluc. All the data of cytokine array analysis and the cytokine array filters are shown in Supplementary Table 2 and Supplementary Figure 1, respectively. *n =* 2. Thirteen cytokines were selected as abundant (>0.3 increase relative to control) and remarkably increased ones (>1.5-fold increase) in co-culture compared with the SUM of mono-cultures. Relative to positive control indicates the relative chemiluminescent (CL) intensity of each cytokine spot of co-culture compared to the average one of positive control spots (see Supplementary Figure 1). Relative to SUM of mono-culture indicates the relative CL intensity of each cytokine dot of co-culture compared to the one of SUM of MLACs and LLC/Fluc mono-cultures. **(B)** The effect of cytokines on LLC growth. LLC/Fluc cells were incubated with indicated concentrations of rCXCL1, rCXCL2, or rCXCL5 for 48 hr and the luciferase activity of LLC/Fluc was measured. Relative LLC/Fluc growth represent relative BL intensity of each sample compared to the one of untreated LLC/Fluc (0 ng/mL). Error bars indicate SEM; ^*^, *P*<0.05 vs PBS. *n =* 4. **(C)** Neutralization assay for candidate factors. LLC/Fluc cells were cultured in the presence of co-culture CM with control rat IgG or indicated neutralizing antibody (0.25 μg/ml α-CXCL1, 4.0 μg/ml α-CXCL2, or 2.50 μg/ml α-CXCL5) for 48 hr and the luciferase activity of LLC/Fluc was measured. Relative LLC/Fluc growth represent the relative BL intensity to the one of LLC/Fluc cultured with 2% FBS-RPMI. Error bars indicate SEM; ^*^, *P*<0.05 vs. CM + Rat control IgG. *n =* 4. **(D)** Knockdown of CXCR2 in LLC/Fluc cells using shRNAs. LLC/Fluc expressing empty vector (empty vector) was used as a negative control. mRNA and protein level of CXCR2 were evaluated by qRT-PCR (top) and flow cytometry (bottom), respectively. (Top) Relative expression represents the mRNA level relative to the one in empty vector. Error bars indicate SEM; ^*^, *P*<0.05 vs. empty vector. *n =* 3. (bottom) Mean fluorescent intensity (MFI) of CXCR2 staining of each LLC clone is shown. Error bars indicate SEM; ^*^, *P*<0.05 vs. empty vector. *n =* 3. **(E)** Impact of cytokine receptors on MLACs-induced LLC growth. After 48 hr of co-culture with MLACs carrying the empty vector or shCXCR2, luciferase activity of LLC/Fluc cells was measured. Error bars indicate SEM; ^*^, *P*<0.05 vs. LLC/Fluc mono-culture. *n =* 4. **(F-H)** Cytokine expression in co-culture of LLC with MLACs (F), Mo-MDSC (G), or PMN-MDSC (H). After 48 hr of co-culture, cells were separately collected and labelled with antibodies against CXCL1, CXCL2, and CXCL5, and then analyzed by flow cytometry. Blue, red, magenta, and green histograms indicate LLC, MLACs, Mo-MDSC, and PMN-MDSC respectively. All the experiments were performed at least three times and representative results are shown.

Considering that the MLACs enhanced co-cultured tumor cell growth through membrane inserts (Figure 2E), the growth-promoting effect must be mediated by soluble factors. Thus, the cytokines secreted by the cells in conditioned culture medium (CM) at the bottom of the co-culture (LLC/Fluc + MLACs) and mono-culture (LLC/Fluc or MLACs) chambers were analyzed with cytokine arrays (Supplementary Figure 1). Thirteen cytokines (CCL2, CCL3, CCL4, CCL5, CXCL1, CXCL2, CXCL5, G-CSF, M-CSF, MMP-2, MMP-3, PAI-1, and VEGFA) were abundantly present in the co-culture medium (>0.3 increase relative to the control) and were also increased by >1.5-fold compared to those detected in the mono-culture medium (Figure 3A, Supplementary Table 2). Among these, CXCL1, CXCL2, and CXCL5 can bind to chemokine receptor CXCR2 [38], which is expressed on several melanoma cell lines [39] and mediates signaling to significantly facilitate their growth *in vitro* and *in vivo* [40, 41]. Thus, to evaluate the direct effects of these cytokines on tumor cell growth, LLC/Fluc cells were cultured in a medium containing each cytokine alone. CXCL1, CXCL2, and CXCL5 promoted LLC tumor growth with an increase in concentration (Figure 3B). To confirm whether the growth-promoting effect of MLACs is mediated by these cytokines, LLC/Fluc cells were cultured in the co-culture (LLC/Fluc and MLACs) CM containing a neutralizing antibody specific for each candidate cytokine, which resulted in a significant inhibition of LLC cell growth (Figure 3C). The contributions of CXCL1, CXCL2, and CXCL5 to LLC cell growth were further confirmed by abolishing the growth-promoting effect of MLACs on LLC cells whose CXCR2 expression was suppressed by short hairpin RNAs (shRNAs) specific to CXCR2 (Figure 3D and 3E). Moreover, CXCL1, CXCL2, and CXCL5 could be abundantly secreted by MLACs (Figure 3F), but were only slightly secreted by Mo-MDSCs and PMN-MDSCs (Figure 3G and 3H). These results revealed another clear difference between MLACs and MDSCs.

### MLACs enhance tumor angiogenesis but fail to suppress CTLs and differentiate into TAMs

**Figure 4:**
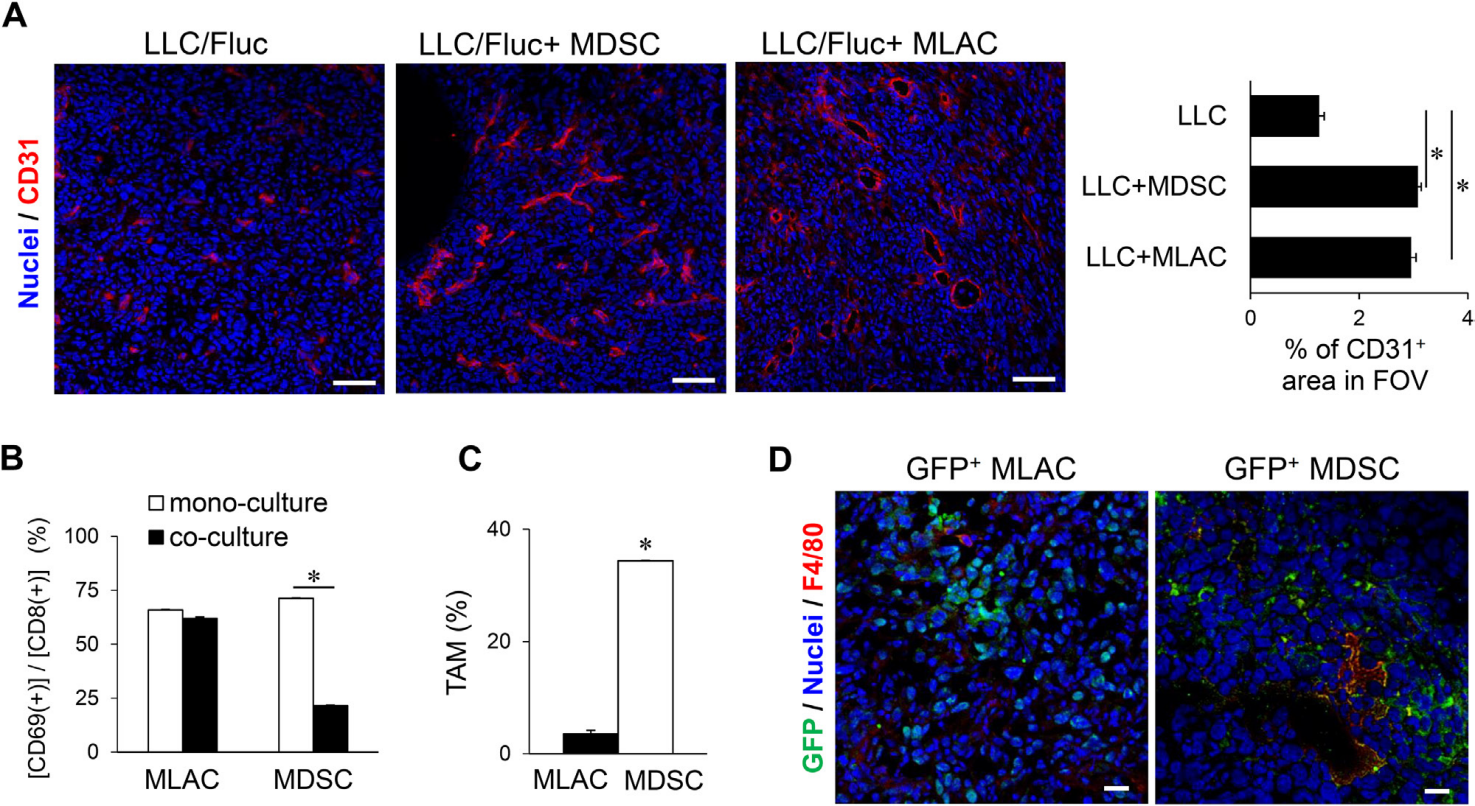
MLACs facilitate angiogenesis in a tumor tissue **(A)** Angiogenesis promotion by MLACs. MLACs or MDSCs were subcutaneously co-injected with LLC/Fluc into B6 Albino mice. Tumor cryosections were prepared at 8 days after transplantation. Left photos show representative immunofluorescence staining images. Red and blue indicate tumor blood vessels (CD31) and nuclei, respectively. Scale bar: 50 μm. Right graph shows the average density of CD31-positive regions of 5 fields of view (FOV) measured by Image J. **(B)** CTL suppression by MLACs. CTLs were co-cultured with or without MLACs or MDSCs for 72 hr, and then the percentage of activated CTLs [CD8^+^ CD69^+^] was measured. Error bars indicate SEM; ^*^, *P*<0.05 vs. mono-culture. *n =* 3. **(C)** MLACs did not differentiate into TAMs *in vitro*. The percentage of mature TAMs [CD11b^+^ F4/80^+^] was measured by flow cytometry after culturing for 5 days. Error bars indicate SEM; ^*^, *P*<0.05 vs. MLACs. *n =* 3. **(D)** Differentiation analysis of MLACs in tumors. MLACs and MDSCs were isolated from LLC subcutaneous tumors of GFP-Tg mice. Then, GFP^+^ MLACs or MDSCs cells were subcutaneously co-injected with LLC/Fluc cells into B6 Albino mice. GFP (green) and F4/80 (red) were detected by immunofluorescence staining of tumor cryosections (day 8). Nuclei are indicated by blue. Scale bar: 20 μm. All the experiments were performed at least three times and representative results are shown.

In the late phase (around day 20) of the co-transplantation experiments (Figure 2A and 2B), the MDSCs showed significant tumor growth-promoting effects, probably through the aforementioned three mechanisms: angiogenesis induction, CTL suppression, and differentiation into TAMs. Thus, for further comparison of these cell populations, we next evaluated the underlying mechanism of the significant tumor-promoting effect of MLACs in the late phase. The cytokine array results showed that the levels of angiogenic factors such as VEGFA, MMP-2, MMP-3, PAI-1, CCL2, CXCL1, CXCL2, and CXCL5 [42–46] were significantly increased in the co-culture medium of MLACs and LLC cells, suggesting angiogenesis induction as one of the mechanisms (Figure 3A and Supplementary Table 2). Indeed, immunohistochemical analysis of LLC tumor tissues injected with or without MLACs revealed that MLACs have strong angiogenic activities comparable to those of MDSCs (Figure 4A). However, co-culture with MLACs did not reduce the CD69^+^ activated CTL ratio in the CD8^+^ T cell population from splenocytes, while MDSCs isolated from the subcutaneous tumor tissue significantly suppressed CTL activation (Figure 4B), indicating that CTL suppression is not the mechanism by which MLACs promote tumor progression in the late phase. Furthermore, the MLACs failed to differentiate into TAMs under the same culture conditions in which MDSCs were able to do so (Figure 4C). Although these results clearly showed that MLACs failed to differentiate into TAMs (Figure 4C), this does not necessarily imply that they are not capable of such differentiation, since the *in vitro* conditions might not be optimized for differentiation. Therefore, we examined the tumors themselves to verify the possibility of *in vivo* differentiation. For this purpose, MLACs or MDSCs isolated from a GFP-Tg mouse were subcutaneously co-injected with LLC cells, the tumor tissue was resected 8 days after co-transplantation, and its cryosections were examined for immunofluorescence staining of GFP and F4/80. Co-localization of F4/80 with GFP was considerably observed in the MDSCs-co-injected tumor sections but was hardly observed in the MLACs-co-injected sections, thereby supporting the *in vitro* results that MLACs are not able to differentiate into TAMs (Figure 4D).

### MLACs enhance MDSCs migration by secreting CCL2/5 and CXCL1/2/5

**Figure 5:**
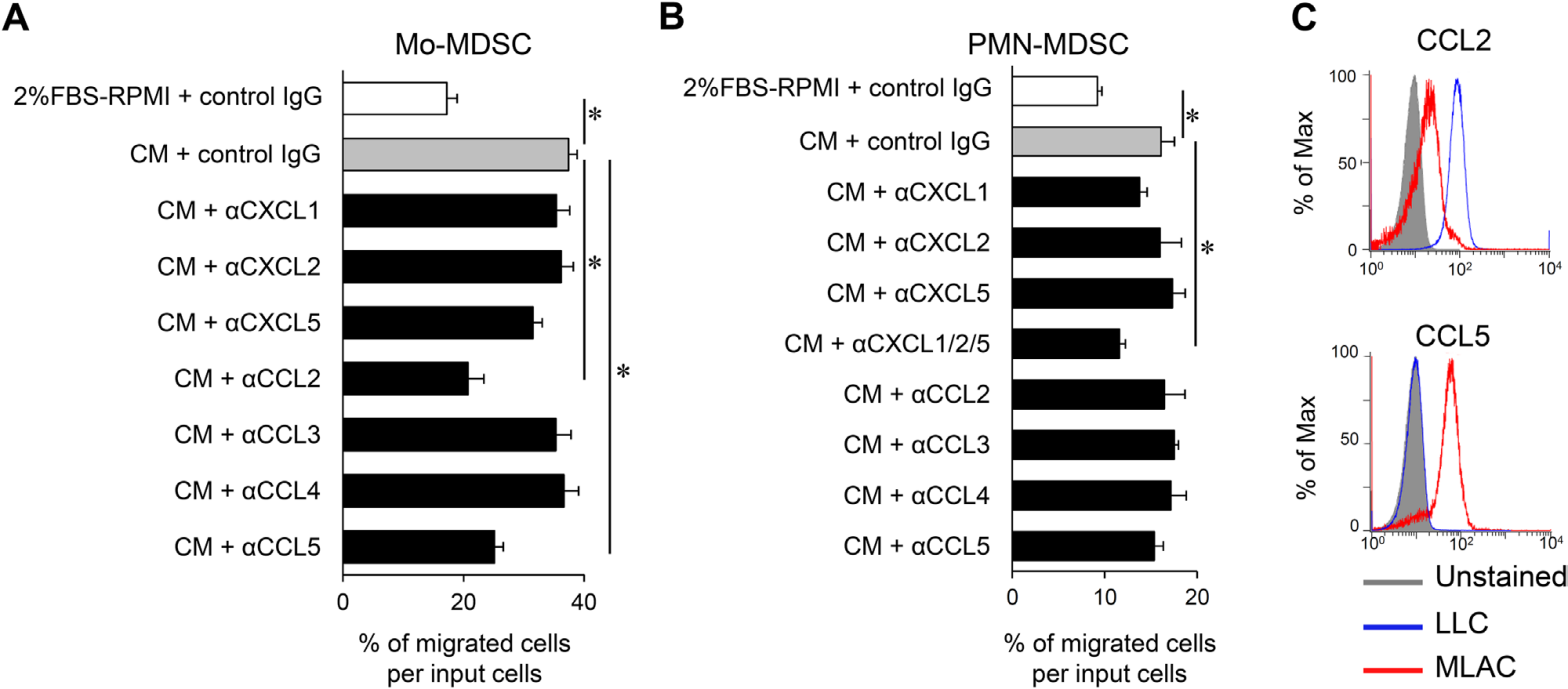
MLACs enhance MDSCs migration by secreting CCL2/5 and CXCL1/2/5 **(A-B)** The effects of neutralization of each candidate factor on recruitment of Mo-MDSCs (A) and PMN-MDSCs (B). PMN-MDSCs or Mo-MDSCs were seeded in a transwell^®^ insert, and co-culture CM containing rat control IgG or indicated neutralizing antibody (0.25 μg/ml α-CXCL1, 4.0 μg/ml α-CXCL2, 2.50 μg/ml α-CXCL5, 3.0 μg/ml α-CCL2, 0.75 μg/ml α-CCL3, 3.0 μg/ml α-CCL4, or 0.50 μg/ml α-CCL5) was added in a lower well. Migration activity is indicated by the percentage of migrated cells per seeded MDSCs (input cells). Error bars indicate SEM; ^*^, *P*<0.05 vs. CM + rat control IgG. *n =* 3. **(C)** Intracellular chemokine expression in LLC and MLACs after co-culture. After 48 hr of co-culture with a membrane insert, LLC and MLACs were separately collected and labelled with antibodies against CCL2 and CCL5 and analyzed by flow cytometry. Blue and red histograms indicate LLC and MLACs, respectively. All the experiments were performed at least three times and representative results are shown.

The results presented thus far demonstrate that MLACs promote tumor progression via a distinct mechanism from that of MDSCs. To explain the tumor growth-promoting effect of MLACs in the late stage (Figure 2B), we hypothesized that MLACs create a tumor microenvironment that is favorable for tumor growth. Among the 13 cytokines shown to be increased by the co-culture of MLACs and LLC cells (Figure 3A), CXCL1, CXCL2, CXCL5, CCL2, CCL3, CCL4, and CCL5 [47–51] are chemokines known to recruit MDSCs. As expected, the CM of the co-culture of MLACs and LLC cells significantly increased the migration of Mo-MDSCs and PMN-MDSCs in an *in vitro* cell migration assay (Figure 5A and 5B). The CM-induced Mo-MDSC migration was dramatically inhibited by blockade of CCL2 or CCL5 using their neutralizing antibodies (Figure 5A), while the CM-induced PMN-MDSC migration was not significantly inhibited by any single neutralizing antibody (Figure 5B). Considering that CXCL1, CXCL2, and CXCL5 can bind to the shared receptor CXCR2 [38], these cytokines may function redundantly and therefore blocking only one of them may not influence the migration of PMN-MDSCs. Indeed, simultaneous blocking of the three cytokines significantly inhibited the migration (Figure 5B). Further intracellular flow cytometry analysis of these chemokines in MLACs after co-culture revealed that MLACs can secrete both CCL2 and CCL5 (Figure 5C) as well as CXCL1, CXCL2, and CXCL5 (Figure 3F), suggesting that MLACs contribute to the recruitment of both MDSC lineages *in vivo*.

### MLACs contribute to MDSCs recruitment into a tumor tissue

**Figure 6:**
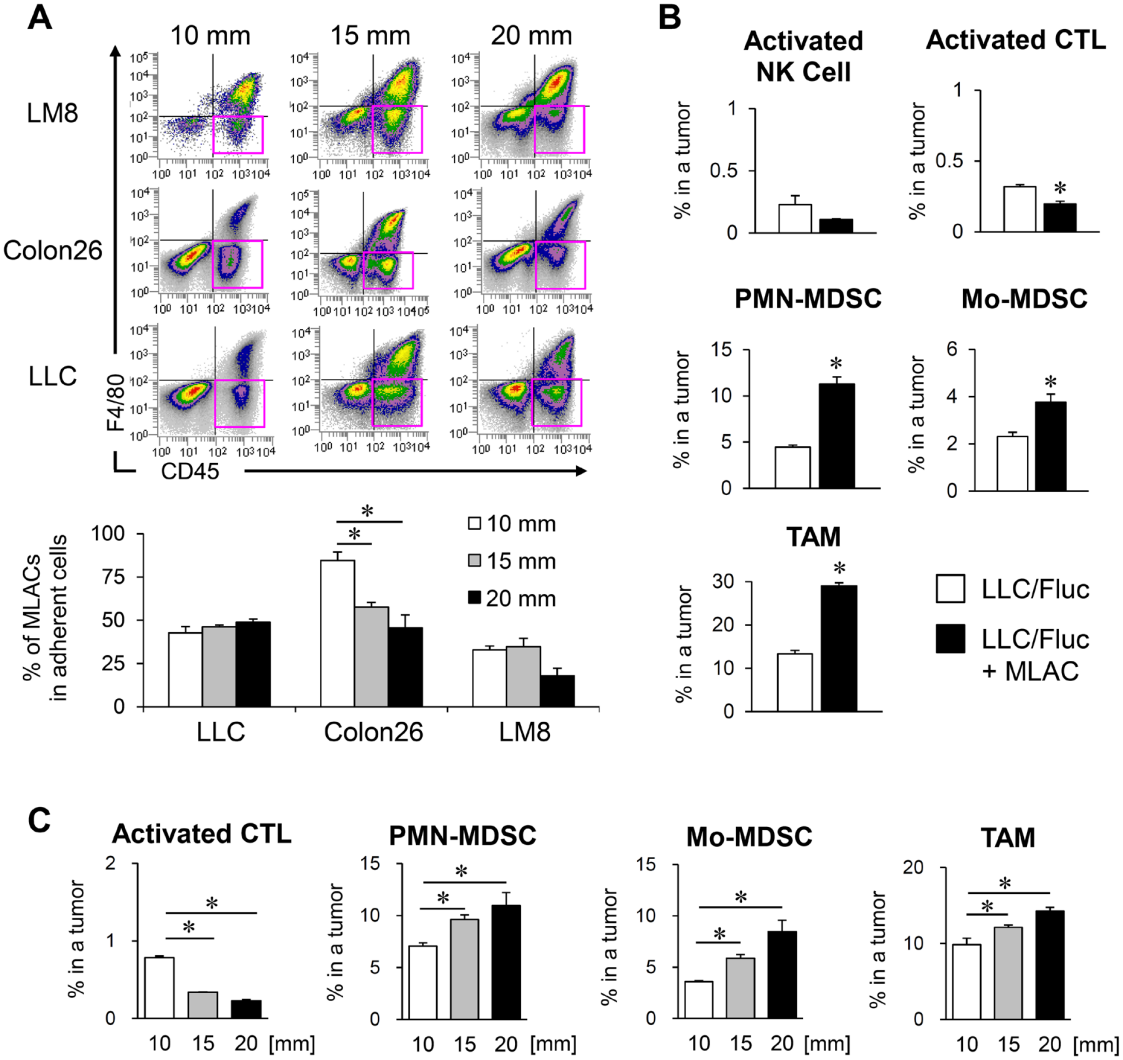
MLACs contribute to MDSCs migration into a tumor tissue **(A)** The frequency of MLACs in tumors. Adherent cells were collected from subcutaneous tumors of three indicated cell lines when their size reached 10, 15, or 20 mm in diameter. The adherent cells were analyzed for their CD45 and F4/80 expressions and pink rectangles indicate MLACs fractions (upper panels). The graph shows the percentages of MLACs in total adherent cells of corresponding size of tumors. Error bars indicate SEM; ^*^, *P*<0.05 vs. the value of 10 mm tumors. *n =* 3. **(B)** Effect of MLACs on the frequencies of host immune cells. Subcutaneous tumors were resected 20 days after co-injection of LLC/Fluc and MLACs. The percentages of activated NK cells [NK1.1^+^ CD69^+^], activated CTLs [CD8^+^ CD69^+^], Mo-MDSCs [CD11b^+^ Gr-1^low^], PMN-MDSCs [CD11b^+^ Gr-1^hi^], and TAMs [CD11b^+^ F4/80^+^] in tumors were analyzed by flow cytometry. Closed and open bars indicate tumors injected with and without MLACs, respectively. Error bars indicate SEM; ^*^, *P*<0.05 vs. LLC/Fluc tumors. *n =* 3. **(C)** The frequencies of activated CTLs, PMN-MDSCs, Mo-MDSCs, and TAMs in LLC tumors with different size. Subcutaneous tumors were resected when their size reached 10, 15, or 20 mm in diameter. The percentages of each cell type were evaluated by flow cytometry. Error bars indicate SEM; ^*^, *P*<0.05 vs. 10 mm tumors. *n =* 5. All the experiments were performed at least three times and representative results are shown.

To search for the generality and timing by which MLACs are recruited into a tumor tissue, we examined tumors of different cancer types and tumor sizes. The frequencies of MLACs were compared among those collected from the syngeneic subcutaneous tumors of LLC cells (C57BL/6), mouse colon cancer Colon26 cells (BALB/c), and mouse osteosarcoma LM8 cells (C3H) when the tumor sizes reached 10, 15, or 20 mm in diameter based on caliper measurements. The MLACs were present at the same or at even higher ratios in smaller-sized tumors independent of tumor type and strain (Figure 6A), suggesting that MLACs would be consecutively recruited into a tumor tissue and generally contribute to tumor progression from an early stage.

To investigate the contribution of MLACs to the recruitment of the infiltrating immune cells to tumors, MLACs were isolated from a GFP-Tg mouse and were subcutaneously co-transplanted with LLC cells. Twenty days after co-injection, the LLC tumors were resected to measure percentages of GFP^−^ host immune cells using flow cytometry. In LLC tumors co-transplanted with MLACs, the frequencies of Mo-MDSCs, PMN-MDSCs, and TAMs significantly increased, while the frequency of activated CTLs significantly decreased compared to that in the LLC-only transplanted tumors (Figure 6B). Both MDSC lineages are progenitor cells of TAMs [11], and TAMs [18] and both MDSC lineages [11] were reported to be able to suppress CTLs. These results demonstrate that the effect of MLACs on the recruitment of both MDSC lineages greatly contributes to tumor progression. To explore the generation of an immunosuppressive network in tumors, we examined the frequencies of activated CTLs, PMN-MDSCs, Mo-MDSCs, and TAMs in subcutaneous tumors of different sizes (Figure 6C). There was a reverse correlation between the frequencies of activated CTLs and tumor size, and positive correlations between the frequencies of other lineages and tumor size (Figure 6C). Considering that MLACs existed at the same ratio or higher in smaller tumors (Figure 6A), these results suggest that MLACs might infiltrate tumors earlier than MDSCs and initiate the development of an immunosuppressive network by recruiting MDSCs to tumors, thereby creating tumor microenvironments favorable for tumor growth.

## DISCUSSION

In this study, we identified and characterized a novel subpopulation of CD11b^+^ Gr-1^+^ myeloid cells, named MLACs that are specifically present in tumor-bearing mice and infiltrate tumors.

**Figure 7:**
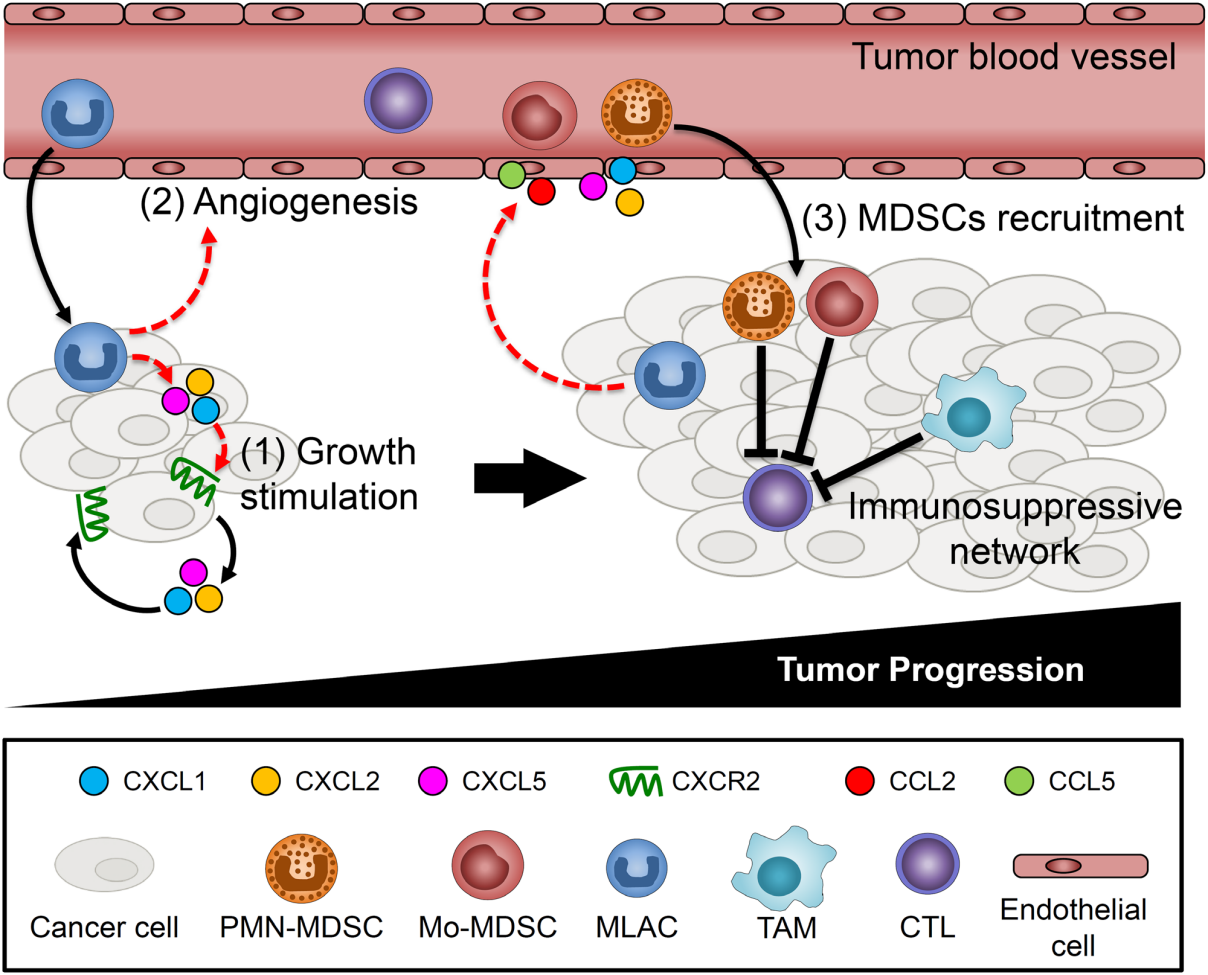
Schematic summary of MLACs functions on tumor progression (1) MLACs are recruited to tumors in an early stage of tumor development and stimulate tumor cell growth through the secretion of CXCL1/2/5 and by activating CXCL1/2/5-CXCR2 autocrine loops of tumor cells. (2) MLACs secrete angiogenic factors to promote tumor angiogenesis. (3) MLACs recruit MDSCs through increase of cytokines in tumors. The recruited MDSCs differentiate to TAMs and together form immunosuppressive network that suppresses CTL and establish an immunosuppressive microenvironment. Dashed red lines indicate MLACs-induced functions and black lines indicate functions/phenomena that closely associated with MLACs functions.

MLACs have both direct and indirect effects on tumor growth (Figure 7). MLACs directly stimulate tumor cell growth by activating the CXCL1/2/5-CXCR2 signaling axis in tumor cells. This direct effect was reflected by the significant increase in tumor growth observed at day 8 in the co-transplantation experiment, which was no longer evident by day 12 when almost all of the transplanted MLACs disappeared. By contrast, the MDSCs did not show such a tumor growth-promoting effect in the early phase. This suggests the lack of a significant direct effect on tumor cells in MDSCs and that MDSCs might need additional support to become fully active and build an immunosuppressive tumor microenvironment. This possibility is consistent with a previous study showing that MDSCs collected from mouse tumor tissues at an early stage could not suppress CTLs [52]. MLACs indirectly stimulate tumor cell growth by enhancing immunosuppression. Although MLACs failed to differentiate into TAMs in tumors and to suppress CTLs *in vitro*, they significantly reduced levels of activated CTLs in tumors (Figure 6B), probably through the recruitment of MDSCs. Further study is required to elucidate the molecular mechanisms of their immunosuppression.

Unlike MDSCs, the MLACs were consistently detected in tumors and showed a tendency toward relatively higher frequencies in smaller tumors (Figure 6A). Although it is difficult to directly compare the frequencies of MLACs and other immune cells in tumors because of the difference in their detection methods, these results strongly suggest that MLACs infiltrate tumors earlier than other immune cells. The results observed in Colon26 cells were particularly remarkable in this regard. Considering that MLACs have the capacity to recruit Mo-MDSCs and PMN-MDSCs through the secretion of CCL2/5 and CXCL1/2/5, respectively, further exploration of the differences in immunosuppressive status among these three cell lines would help to elucidate the significance of MLACs in creating an immunosuppressive network.

Furthermore, the cytokine array analysis suggested that the levels of G-CSF and M-CSF would be increased by the MLACs–tumor cell interaction in tumors. G-CSF promotes the survival and activation of MDSCs through the STAT3 signaling pathway [53] and systemically increases the number of PMN-MDSCs [54, 55]. M-CSF facilitates the differentiation of MDSCs into DCs [56] and TAMs [57]. Based on these findings, we hypothesize that MLACs may function as an initiator of an immunosuppressive network to create a more favorable microenvironment for tumor cell growth and malignant progression (Figure 7).

Because MLACs were only found in tumor-bearing mice, they could serve as excellent cancer-specific markers. However, the MLACs were isolated independent of the detection of cell surface markers, and there is no specific marker for this subpopulation at present. Furthermore, MLACs themselves seem to consist of several subpopulations with different expression levels of Gr-1, Ly6G, and Ly6C. Thus, identification of specific markers to identify the whole population and subpopulations would be required to gain more detailed knowledge on MLACs. Without specific markers, it is difficult to find a clue to answer the most important question for clinical translation: whether or not cells corresponding to mouse MLACs exist in humans. Because there would be an insufficient amount of circulating MLACs available from solid tumor biopsy samples of patients to recognize them based only on determination of their adherent nature, specific markers will be needed for their effective isolation from clinical samples. Therefore, the next immediate aim should be to identify specific markers for MLACs. This may be addressed using single-cell RNA sequencing and proteomics analysis of cell surface proteins from tumor-bearing mice. Recognition of mouse MLAC markers would help to identify specific markers for human MLACs, if they exist, which could then become potential therapeutic targets to prevent or delay malignant progression.

The fact that MLACs were detected in the bone marrow, spleen, and peripheral blood of tumor-bearing mice indicates that these cells may be generated in and recruited from the bone marrow and/or spleen to the tumor tissue through the circulation. The specific origin of MLACs might be identified by utilization of a mouse model expressing the photoconvertible protein Kikume Green-Red, which changes color from green to red upon violet light-irradiation [58]. By investigating the color of MLACs isolated from the tumors of Kikume Green-Red-Tg mice whose bone marrow or spleen cells have been irradiated with violet light in advance, we may be able to determine the tissues from which MLACs originate. The present analysis of cell surface protein expression on MLACs revealed that they did not express CSF-1R and CX_3_CR1, but did express CCR2. This suggests that MLACs may be recruited by tumor tissue-derived CCL2, which is known to be mainly produced by monocyte/macrophages [59, 60], suggesting that CCL2 may play a role to maintain a positive feedback loop to establish the immunosuppressive network in tumor tissues. The soluble factors that recruit MLACs to a tumor tissue should be further investigated to best understand the roles of MLACs as an initiator of the immunosuppressive network.

MDSCs and TAMs are proven to have various impacts on tumor progression, such as inducing angiogenesis, immunosuppression, and tumor metastasis. Considering that MLACs have capacities to induce the recruitment of MDSCs and the accumulation of both MDSCs and TAMs in a tumor tissue, a novel treatment strategy of eliminating MLACs may help to weaken the immunosuppressive network activities, leading to a more favorable tumor microenvironment for host immunity and immune checkpoint therapy.

## MATERIALS AND METHODS

### Mice

B6(Cg)-*Tyr*^*c-2J*^*/J* (B6 albino) mice and GFP-Tg were maintained in both Kyoto University and Tokyo Institute of Technology. BALB/c and C3H mice were obtained from Charles River Laboratory Japan (Yokohama, Japan). All mice used were littermates or age-matched (between 6 and 11 weeks of age) males. They were provided access to food and water *ad libitum*, and were housed in the animal facilities at Tokyo Institute of Technology.

All the experimental procedures using mice were approved by the Animal Experiment Committees of Tokyo Institute of Technology (authorization number D2014005) and Kyoto University (authorization number S-16-4-2), and carried out in accordance with relevant national and international guidelines.

### Cells and culture conditions

The murine lung carcinoma cell line LLC and murine colon carcinoma cell line Colon26 were obtained from ATCC (Maryland, USA). The murine osteosarcoma cell line LM8 was gifted from Dr. Hideki Yoshikawa (Osaka University, Osaka, Japan). LLC and LM8 cells were maintained with 5% fetal bovine serum-Dulbecco's Modified Eagle's medium (FBS-DMEM) (Nacalai Tesque, Kyoto, Japan) supplemented with penicillin (100 units/ml) and streptomycin (100 mg/ml), and Colon26 cells were maintained with 10% FBS-RPMI-1640 medium (FBS-RPMI) (Nacalai Tesque) supplemented with penicillin (100 units/ml) and streptomycin (100 mg/ml). The cells were cultured in 5% CO_2_ incubator at 37°C and regularly checked for mycoplasma contamination by a mycoplasma check kit (Lonza, Basel, Switzerland). All the cell lines were independently stored and recovered from the original stock every time for each experiment.

### Plasmid construction

To construct a plasmid to constitutively express mKO2-Rluc8.6, cDNA of CMV promoter-multi cloning site (MCS)-poly A was amplified from pcDNA3.1 plasmid (Invitrogen, California, USA) and inserted into Addgene plasmid #26553 (Addgene, Massachusetts, USA) to obtain pT2/CMV-MSC-SVNeo. Next, to construct pcDNA/mKO2-Rluc8.6, cDNAs of mKO2 and Rluc8.6 were amplified from Addgene plasmid #67661 (Addgene) and pGEX/PTD-ODD-Rluc8.6 [61], respectively. Then, amplified mKO2-Rluc8.6 cDNA from pcDNA/mKO2-Rluc8.6 was ligated into pT2/CMV-MCS-SVNeo using In-Fusion^®^ HD Cloning Kit (Clontech, California, USA) to obtain pT2/CMV-mKO2-Rluc8.6-SVNeo.

### Isolation of cancer cell lines stably expressing Fluc or Rluc reporters

LLC/Fluc, Colon26/Fluc, and LM8/Fluc cells were isolated after transfection with plasmid pEF/luc [62], by the calcium phosphate method [63]. LLC/mKO2-Rluc8.6 was isolated after co-transfection with Sleeping Beauty transposon-based plasmid pT2/mKO2-Rluc8.6 and the SB100X sleeping beauty transposase plasmid pCMV-SB100X (Addgene) by electroporator (NepaGene Co., Chiba, Japan). The transfected cells were selected in medium containing G418 (ThermoFisher Scientific, California, USA). G418-resistant colonies were isolated and established as clones. The luciferase activity was measured using Luciferase Assay Kit (Promega, Wisconsin, USA) and the clones with high luciferase activity were used here.

### Subcutaneous xenograft model

The cell suspensions of LLC/Fluc (3.0 × 10^5^ cells), LLC/mKO2-Rluc8.6 (3.0 × 10^5^ cells), Colon26/Fluc (3.0 × 10^5^ cells), or LM8/Fluc (3.0 × 10^5^ cells) in phosphate-buffered saline (PBS) were mixed with an equal volume of Geltrex^®^ (Invitrogen) and subcutaneously injected into the hindlimbs of syngenic mice. Mice with subcutaneous tumors of 10–20 mm in diameter were used for experiments.

### Flow cytometry and cell sorting

Single cell suspensions from tumor tissues, spleens, or bone marrow were incubated in PBS containing α-CD16/32 (clone 93, Biolegend, California, USA, 1:200) at 4°C for 15 min to block Fc receptors prior to staining cells with fluorochrome-labeled antibodies at 4°C in the dark for 25 min in 2% FBS-PBS. The antibodies used for surface marker staining are as follows: α-CCR2 (clone #475301, R&D Systems, Minnesota, USA, 1:50), α-CD8a (clone 53-6.7, Biolegend, 1:100), α-CD11b (clone M1/70, Biolegend, 1:200), α-CD11c (clone N418, Biolegend, 1:200), α-CD45 (clone 30-F11, Biolegend, 1:200), α-CD68 (clone FA-11, Biolegend, 1:200), α-CD69 (clone H1.2F3, Biolegend, 1:100), α-CSF-1R (clone AFS98, Biolegend, 1:200), α-CX_3_CR1 (polyclonal, R&D Systems, 1:200), α-F4/80 (clone CI:A3-1, AbD Serotec, North Carolina, USA, 1:50), α-Gr-1 (clone RB6-8C5, Biolegend, 1:200), α-Ly6C (clone HK1.4, Biolegend, 1:200), α-Ly6G (clone 1A8, Biolegend, 1:200), α-NK1.1 (clone PK136, Biolegend, 1:100), α-CXCR2 (clone # 242216, R&D Systems, 1:100), α-c-Kit (clone 2B8, Biolegend, 1:200), α-MHC-II (clone M5/114.15.2, BD, 1:400), α-CD34 (clone RAM34. BD, 1:100), α-Siglec-F (clone RNM44N, eBioscience, San Diego, USA, 1:200), α-FcεRIα (clone MAR-1, Biolegend, 1:200). For intracellular cytokine staining, cells were resuspended in fixation-permeabilization buffer (FoxP3/Transcription Factor Staining kit from eBioscience) and incubated at 4°C in the dark for 25 min, then cells were washed with Permeabilization Buffer (eBioscience) and labeled with antibodies to cytokines at 4°C in the dark for 25 min in Permeabilization Buffer (eBioscience). The antibodies used for intracellular staining are as follows: α-CCL2 (clone 2H5, Biolegend, 1:200), α-CCL5 (2E9/CCL5, Biolegend, 1:200), α-CXCL1 (polyclonal, R&D Systems, 1:200), α-CXCL2 (polyclonal, R&D Systems, 1:200), α-CXCL5 (polyclonal, R&D Systems, 1:400). Multiple-color flow cytometric analysis was performed using a flow cytometer iCyt ec800 (Sony Biotechnology, California, USA). For cell sorting, prepared cells were sorted using a fluorescence activated cell sorter FACSAria (Becton, Dickinson and Company, New Jersey, USA).

### Isolation of MLACs from tumors

Subcutaneous tumors of 10-20 mm in diameter were resected, well-minced and digested in 2% FBS-RPMI containing 2.6 U Liberase DH (Roche Applied Science, Indiana, USA) at 37°C for 60 min, and then sequentially passed through four different pore size (500, 250, 100, and 40 μm) strainers (Greiner bio-one, Kremsmünster, Austria) to obtain a single-cell suspension. To lyse red blood cells, the cells were collected by centrifugation, suspended in PharmLyse solution (Becton, Dickinson and Company) and incubated for 10 min at room temperature. Then the cells were seeded into a dish with 2% FBS-RPMI at 37°C for 30 min to allow attaching to a plastic surface and extensively washed three times with PBS containing 2 mM EDTA, and adherent cells strongly attach to plastic surfaces were collected with a cell scraper. CD11b^+^ F4/80^−^ cells were sorted from single cell suspensions of the adherent cells and used as MLACs.

### Investigation of the presence of MLACs in normal tissues

For detection of MLACs from normal tissues, the spleen, peripheral blood, and bone marrow of femurs and tibias were removed from mice bearing tumors with 15-20 mm in diameter and cells isolated from whole tissues were then passed through 40 μm cell strainer (Greiner bio-one) to obtain a single-cell suspension. The single-cell suspensions were then collected by centrifugation, suspended in PharmLyse solution (Becton) and incubated for 10 min at room temperature. Then the cells were seeded into a dish with 2% FBS-RPMI at 37°C for 30 min to allow attaching to a plastic surface and extensively washed three times with PBS containing 2 mM EDTA, and adherent cells strongly attach to plastic surfaces were collected with a cell scraper. Then, expression of CD45 and F4/80 in the adherent cells were analyzed for the presence of MLACs.

### Cell morphology analysis

The cell pellet of MLACs, TAMs, Mo-MDSCs, or PMN-MDSCs (1.0 × 10^4^ cells each) were smeared onto glass slides and air-dried. The smear was then stained with May-Grünwald/Giemsa solutions (Sigma Aldrich, Missouri, USA) as described by the manufacturer's protocol and photos were taken under light microscopy.

### RNA isolation and qRT-PCR

Total RNA was isolated from cell pellets using RNeasy^®^ Mini Kit (Qiagen, California, USA) as described by the manufacturer's protocol. One μg of total RNA was reverse-transcribed with Oligo(dT)_20_ Primer (Toyobo Co., Osaka, Japan) and ReverTra Ace^®^ (Toyobo Co.). The qRT-PCR was conducted using THUNDERBIRD^®^ SYBR^®^ qPCR mix (Toyobo Co.) in a LightCycler^®^ 2.0 (Roche Applied Science). All reactions were performed in triplicate. The relative amount of mRNA was normalized against *Actb*. The primers used are listed in the Supplementary Table 3.

### Isolation of MDSCs

Preparation of tumor cell suspension and lysis of red blood cells were performed as described in isolation of MLACs. After removing lysed red blood cells, CD11b^+^ Gr-1^+^ cells, CD11b^+^ Ly6C^low^ Ly6G^+^ cells, and CD11b^+^ Ly6C^high^ Ly6G^−^ cell were sorted from the single cell suspension and used as MDSCs, PMN-MDSCs, and Mo-MDSCs, respectively.

### Generation of bone marrow-derived dendritic cell (BMDC)

Bone marrow cells were differentiated into dendritic cells as described previously [64]. Briefly, cells were collected from the marrow of femurs and tibias of B6 Albino mice and the cells (2.0 × 10^6^) were cultured in 10 ml 10% FBS-RPMI containing 20 ng/ml GM-CSF (Peprotech) and 50 μM 2-mercaptoethanol. After 4 and 7 days of culture, the non-adherent cells were collected and cultured in fresh GM-CSF-containing medium. After additional 10 days of culture, the supernatant was harvested and the adherent cells were incubated with 5 ml 2 mM EDTA at 37°C for 5 min. After gentle agitation, the floating cells were collected as BMDCs and washed two times with PBS.

### *In vivo* bioluminescence (BL) imaging

LLC/Fluc cells (3.0 × 10^5^) were subcutaneously injected into the hindlimb of 6-11-week-old B6 albino mice with or without of MLACs, Mo-MDSCs, or PMN-MDSCs (4.0 × 10^5^ cells each). Tumor-bearing mice were intraperitoneally injected with 150 μl of 100 μg/ml D-luciferin solution (Promega) and imaged at 20 min post-injection in an *in vivo* photoncounting device IVIS^®^-spectrum (Perkin Elmer, Illinois, USA). The following conditions were used for image acquisition: exposure time = 2 min, binning = medium: 8, field of view = 22.5 × 22.5 cm, and f/stop = 1. The minimum and maximum photons/s/cm^2^/sr of each image is indicated in each Figure by a rainbow bar scale.

### Immunohitochemical analysis

Tumor tissues were harvested and immediately frozen in optimum cutting temperature (O.C.T.) compound (Sakura Finetek Japan, Tokyo, Japan). Frozen tumors were 10-μm cryosectioned using a Microm HM560MV cryostat (ThermoFisher Scientific) and fixed in 4%-Paraformaldehyde Phosphate Buffer Solution (Nacalai Tesque) for 10 min at room temperature. The sections were incubated in blocking buffer (3% BSA in PBS) for 1 hour at room temperature, and then incubated with primary antibodies diluted in blocking buffer overnight at 4 °C. After washing with PBS three times for 5 min, the samples were incubated for 1 hr at room temperature in the dark with fluorochrome-conjugated secondary antibodies. Nuclei were stained with Bisbenzimide H33342 Fluorochrome Trihydrochloride (Nacalai Tesque). Sections were washed with PBS three times for 5 min, and then mounted coverslips on slides using Fluoromount (Diagnostic BioSystems, California, USA). Images were obtained on an LSM780 confocal microscopy (Carl Zeiss, Oberkochen, Germany). Primary antibodies used are as follows: α-CD11c (clone N418, Biolegend, 1:50), α-F4/80 (clone BM8, Biolegend, 1:50), α-GFP (polyclonal, Abcam, Massachusetts, USA, 1:250), α-CD31 (clone MEC 13.3, BD, 1:50), and α-Rluc (polyclonal, MBL international, Massachusetts, USA, 1:100). Fluorochrome-labeled secondary antibodies (ThermoFisher Scientific) were used at 1:1000 dilutions.

### Lifetime of MLACs in a tumor tissue

MLACs were isolated from LLC-tumor-bearing GFP-Tg mice, and the GFP^+^ MLACs (4.0 × 10^5^ cells) were injected into the hindlimbs of 6-11-week-old B6 Albino mice with LLC/mKO2-Rluc8.6 cells (3.0 × 10^5^ cells). On days 8, 12, and 20 after co-injection, tumor tissues were resected and existence of MLACs in tumor tissues were observed under a confocal fluorescent microscope (on an LSM780, Carl Zeiss) after immunostaining for GFP.

### *In vitro* co-culture assay

Boyden-chamber assays were performed using a 6.5-mm transwell^®^ with 0.4-μm pore membrane insert (Corning, New York, USA). LLC/Fluc cells (2.4 × 10^4^ in 600 μL medium) were seeded into the bottom chamber, and MLACs, Mo-MDSCs, or PMN-MDSCs (3.2 × 10^4^ in 100 μL medium) were seeded in the upper chamber of the transwell^®^. After 48 hr of culture at 37°C, the tumor cells were lysed with Passive Lysis Buffer (Promega) and the luciferase activity was measured using a Luciferase Assay Kit (Promega) with a luminometer GL-210A (Microtec Co., Ltd, Chiba, Japan).

### Cytokine array

After 48 hr of culture in a 6.5-mm transwell^®^ with 0.4-μm pore membrane insert (Corning), the CM of the bottom chamber of co-culture and mono-culture of MLACs and LLC was collected, centrifuged at 400 *g* for 10 min at 4°C, and filtered with 0.22 μm cell strainer (Pall Corporation, New York, USA) to completely remove residual cells. Mouse XL cytokine arrays (R&D Systems) were incubated with each CM and processed according to the manufacturer’s instructions. The chemiluminescent signals were detected using ImageQuant LAS 4000 and quantified with ImageQuant TL software (GE Healthcare Life Sciences, Pennsylvania, USA).

### Isolation of LLC cells stably expressing shRNA against *Cxcr2*

The shRNA sequences against mouse *Cxcr2* (#1; 5’-GGGAGAATTCAAGGTGGATAA-3’ and #2; 5’-GCTATGAGGATGTAGGTAACA-3’) were designed using Block-iT program (Invitrogen) and cloned into the pSUPER.neo+GFP vector (OligoEngine, Washington, USA) to construct a *Cxcr2* shRNA-expressing plasmid. LLC was transfected with the plasmid by an electroporator (NepaGene Co.) and cultured in medium containing 500 μg/mL G418 Sulfate (ThermoFisher Scientific) to establish stable cell clones expressing *Cxcr2* shRNA. The expression levels of protein and mRNA in the clones were assayed by flow cytometry and qRT-PCR, respectively, and the clone with the lowest *Cxcr 2* expression was used here. As a control, LLC cells were transfected with empty pSUPER.neo+GFP vector (OligoEngine).

### Suppression of T-cell proliferation

CD8^+^ CTLs (5.0 × 10^5^ cells) sorted from splenocytes were cultured with or without MLACs or MDSCs (4.0 × 10^5^ cells each) in 10% FBS-RPMI containing 0.5 μg/ml α-CD3ε (clone 145-2C11, Biolegend) and 3.0 μg/ml α-CD28 (clone 37.51, Biolegend) in 6-cm dishes. After 72 hr of culture, the cells were harvested and the percentage of activated (CD69^+^) CTL cells within the CD8^+^ cell population was analyzed by flow cytometry.

### Preparation of tumor tissue conditioned medium (TTCM)

When subcutaneous tumors reach 15-20 mm in diameter, tumor tissues were well-minced and digested in 2% FBS-RPMI containing 2.6 U Liberase DH (Roche Applied Science) at 37°C for 60 min. The cell pellet was resuspended in serum-free RPMI-1640 and cultured at 37°C. After 48 hr of culture, the culture medium was collected and the supernatant was centrifuged at 400 *g* for 10 min at 4°C, and filtered with 0.22 μm pore membrane (Pall Corporation) to prepare TTCM.

### *In vitro* differentiation assay

MLACs and MDSCs were cultured in 10% FBS-RPMI 1640 containing 25% LLC TTCM with 10 ng/ml rGM-CSF (Peprotech). Cells were collected after 5 days of culture, and percentages of F4/80^+^ TAM were analyzed by flow cytometry.

### *In vivo* differentiation analysis

MLACs and MDSCs were isolated from LLC-tumor-bearing GFP Tg mice, and the GFP^+^ MLACs or MDSCs (4.0 × 10^5^ cells each) were injected into the hindlimbs of 6-11-week-old B6 Albino mice with LLC/Fluc cells (3.0 × 10^5^). Tumor tissues (day 8) were resected and the cryosections were examined by immunohistochemical analysis using α-F4/80 antibody.

### *In vitro* migration assay

PMN-MDSCs or Mo-MDSCs were sorted from LLC-tumor-bearing GFP Tg mice, and the GFP^+^ PMN-MDSCs or GFP^+^ Mo-MDSCs were seeded in a 6.5-mm transwell^®^ with 8-μm pore membrane inserts (Corning) in serum-free RPMI. The inserts were placed in 24-well plates with 2% FBS-RPMI containing control rat IgG (R&D Systems), or co-culture CM containing control rat IgG (R&D Systems) or neutralizing antibody against CXCL1, CXCL2, CXCL5, CCL2, CCL3, CCL4, or CCL5 (R&D Systems). After 12 hr, the number of cells migrated to the lower well were counted by a flow cytometer iCyt ec800 (Sony Biotechnology).

### Statistical analysis

The statistical significance between values was determined by Student’s t-test. All data were expressed as the mean ± standard error of the mean (SEM). Probability values 0.05 or less were considered significant.

## SUPPLEMENTARY MATERIALS FIGURES AND TABLES




