# Supplementary material for: Novel adherent CD11b+ Gr-1+ tumor-infiltrating cells initiate an immunosuppressive tumor microenvironment
Source: Oncotarget. 2018 Jan 29;9(13):11209–26. doi: 10.18632/oncotarget.24359 (PMC5834266; doi:10.18632/oncotarget.24359)
Supplement: Supplementary file 1 [file oncotarget-09-11209-s001.pdf]

Novel adherent CD11b^{+} Gr-1^{+} tumor-infiltrating cells initiate an immunosuppressive tumor microenvironment

SUPPLEMENTARY MATERIALS

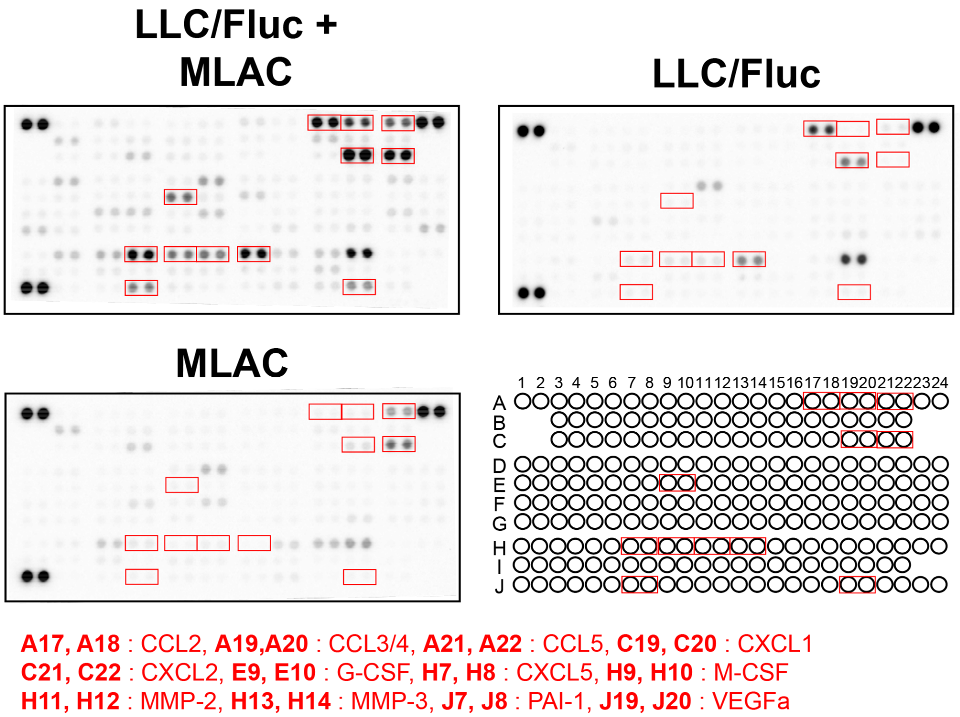

Supplementary Figure 1: Cytokine assay analysis. Cytokine array filters spotted with antibodies against 111 different cytokines were incubated with the mediums of co-culture (LLC/Fluc + MLACs) and mono-culture (LLC/Fluc or MLACs) and treated as described in Cytokine array of Method section and resultant filters are shown. Spots marked with red rectangles indicate 12 cytokines listed in Figure 3A and the name of cytokines corresponding to marked spots are listed below. Spots of position A1, A2, A23, A24, J1, and J2 indicate positive control.

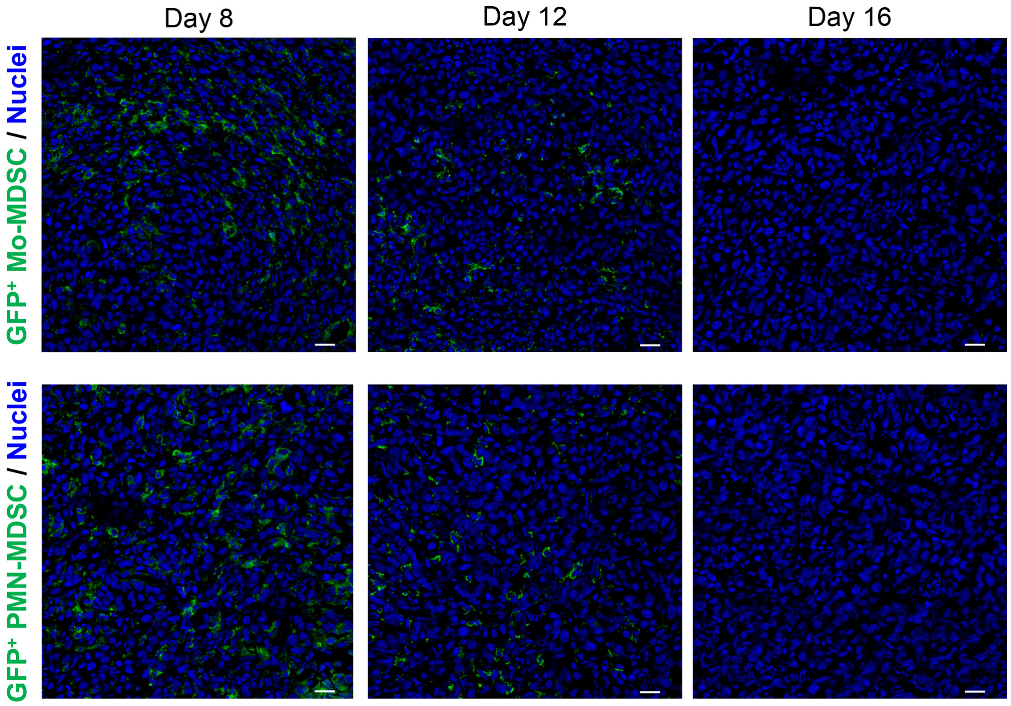

Supplementary Figure 2: Lifetime analysis of Mo-MDSCs and PMN-MDSCs. Mo-MDSCs and PMN-MDSCs were separately isolated from LLC subcutaneous tumors of GFP-Tg mice, and subcutaneously co-injected with LLC/mKO2-Rluc8.6 to B6 albino mice. Subcutaneous tumors were resected on days 8, 12, and 16 after co-injection. GFP (green) was detected by immunofluorescence staining of tumor cryosections. Nuclei are indicated by blue. Representative immunofluorescence staining images of the LLC tumors. Scale bar: 20 μm.

Supplementary Table 1: List of the known markers of myeloid cells detected in tumors

	F4/80	CSF-1R	CD68	CCR2	CX3CR1	CD11c	Ly6C	Ly6G	FcεRIα	c-Kit	CD34	Siglec-F
MLAC	-	-	-	+	-	-	hi/low	+/-	-	-	+	-
TAM	hi	+	+	+	nd	low	+	-	-	-	-	-
Monocyte	low	+	+	hi/low	low/hi	low	hi/low	-	-	-	-	-
Dendritic cell	+	nd	nd	nd	nd	hi	+	-	-	-	-	-
Mo-MDSC	low	+	nd	+	nd	nd	hi	-	-	-	+/-	-
PMN-MDSC	-	+/-	nd	+	nd	nd	low	+	-	-	+/-	-
Eosinophil	+	-	nd	nd	-	-	low	-	-	-	-	+
Basophil	nd	nd	nd	nd	nd	+	nd	nd	+	-	-	-
Mast Cell	nd	nd	nd	nd	nd	-	nd	nd	+	+	+	-

Supplementary Table 2: List of raw data of 111 cytokines tested by cytokine array

See Supplementary File 1

Supplementary Table 3: List of primers used for qRT-PCR

Gene name	Primer sequences
Actb	Forward: 5’-GGCTACAGCTTCACCACCAC-3’<br>Reverse: 5’-TACTCCTGCTTGCTGATCCAC-3’
S100a8 (Calgranulin A)	Forward: 5’-GTCCTCAGTTTGTGCAGAATATAAA-3’<br>Reverse: 5’-TGAGATGCCACACCCACTTT-3’
Mafb	Forward: 5’-GCAACGGTAGTGTGGAGGAC-3’<br>Reverse: 5’-TTCAGGCGGATCACCTCGT-3’
Zbtb46	Forward: 5’-GCCGAGACTCAAATGTAGACCT-3’<br>Reverse: 5’-GGCCCAAGTAGCTGGTTTCT-3’
Cebpa (Cebp alpha)	Forward: 5’-GAACAGCAACGAGTACCGGG-3’<br>Reverse: 5’-GCGGTCATTGTCACTGGTCA-3’
Cebpb (Cebp beta)	Forward: 5’-ATCCGGATCAAACGTGGCTG-3’<br>Reverse: 5’-CCCGCAGGAACATCTTTAAGTGA-3’
Mertk	Forward: 5’-AGCGCAGGGACTTACAAAGAG-3’<br>Reverse: 5’-TTCACTGGCTCACTGAAGGG-3’
Irf8	Forward: 5’-ACACCATTCAGCTTTCTCCCAG-3’<br>Reverse: 5’-CGGCCCATACAACTTAGGCA-3’
Cxcr2 (Il-8r)	Forward: 5’-CCTGCCTCAGACTTTTGGCT-3’<br>Reverse: 5’-GAAGACAAGGACGACAGCGA-3’
